# Program Directors’ Assessments of US Medical Graduates’ Transition to Residency

**DOI:** 10.1001/jamanetworkopen.2024.54048

**Published:** 2025-01-09

**Authors:** Douglas Grbic, Dorothy A. Andriole, Lindsay Roskovensky, Mark Speicher, Keith A. Horvath, Lisa Howley

**Affiliations:** 1 Medical Education Research, Association of American Medical Colleges, Washington, DC; 2 Data Operations and Services, Association of American Medical Colleges, Washington, DC; 3 American Association of Colleges of Osteopathic Medicine, Bethesda, Maryland; 4 Clinical Transformation, Health Care Affairs, Association of American Medical Colleges, Washington, DC; 5 Transforming Medical Education, Association of American Medical Colleges, Washington, DC

## Abstract

**Importance:**

Medical school graduates across specialties should be prepared for the start of postgraduate year 1 (PGY-1). Assessments by program directors (PDs) may offer insight to differences in preparedness across medical specialties.

**Objective:**

To investigate whether PD assessments of their PGY-1 residents’ performance during the transition to residency differed by specialty category.

**Design, Setting, and Participants:**

This retrospective, cross-sectional study used Resident Readiness Survey (RRS) data collected from PDs from 2020 through 2023. Annually, eligible PDs (ie, PDs training PGY-1 residents who had graduated from US medical schools included in the RRS process) were invited to respond. A deidentified, individual-level database of PGY-1 residents for whom PDs were asked to complete standardized RRSs was created to examine the association between graduate medical education (GME) program specialty category (among other variables) and PD response to the RRS item “During the transition to GME (0-6 months of PGY-1 year), did this resident meet overall performance expectations?” The response options included exceeded, met, and did not meet expectations. Analyses included χ^2^ tests to measure bivariate associations and multivariable logistic regression analyses to test independent associations with this outcome.

**Exposures:**

PGY-1 in a specialty program during the first 6 months of GME.

**Main Outcomes and Measures:**

The main outcomes of interest were PD assessment of PGY-1 residents’ overall performance (dichotomized as met or exceeded expectations vs did not meet expectations), PGY-1 year, and GME program specialty category.

**Results:**

The PD RRS response rate varied by year (1786 of 2847 [62.7%] for 2020-2021, 2107 of 3406 [61.9%] for 2021-2022, and 2835 of 3659 [77.5%] for 2022-2023; *P* < .001). Responding PDs provided overall performance ratings for 29 461 PGY-1 residents, including 934 (3.2%) who did not meet expectations and 28 527 (96.8%) who met or exceeded expectations. The percentage of PGY-1 residents who did not meet expectations varied by specialty category (range, 11 of 1482 [0.7%] for transitional year to 235 of 3775 [6.2%] for family medicine; *P* < .001). In multivariable regression, compared with internal medicine, the odds of residents not meeting (vs met or exceeding) expectations were higher for PGY-1 residents in family medicine (adjusted odds ratio [AOR], 2.09 [95% CI, 1.70-2.58]), general surgery (AOR, 2.05 [95% CI, 1.62-2.58]), and obstetrics and gynecology (AOR, 1.64 [95% CI, 1.24-2.15]); in contrast, the odds were lower for PGY-1 residents in other surgical specialties (AOR, 0.60 [95% CI, 0.42-0.84]), other nonsurgical specialties (AOR, 0.61 [95% CI, 0.44-0.85]), and transitional year (AOR, 0.22 [95% CI, 0.12-0.42]) vs internal medicine.

**Conclusions and Relevance:**

Findings from this cross-sectional study indicated that most PGY-1 residents met or exceeded PD expectations. Specialty-specific interventions may further optimize the transition for all US medical graduates.

## Key Points

**Question:**

Do program director assessments of postgraduate year 1 (PGY-1) trainees’ performance during the transition to residency differ by specialty category?

**Findings:**

This multiyear cross-sectional study of 29 461 PGY-1 residents who graduated from US medical schools found that 3.2% did not meet the overall expectations of program directors. However, this percentage varied significantly by specialty category.

**Meaning:**

Findings from this cross-sectional study indicated that most PGY-1 residents met or exceeded their program director’s expectations; specialty-specific interventions may further optimize the transition for all US medical graduates.

## Teaser

This cross-sectional study uses program director responses on the Resident Readiness Survey to assess the performance of postgraduate year 1 residents during the transition to residency and whether that assessment differs by specialty.

## Introduction

US medical graduates enter residency training in a range of specialties for which they may be variably prepared for the responsibilities that they assume at the start of graduate medical education (GME). National aggregated data collected from program directors (PDs) responsible for training postgraduate year 1 (PGY-1) residents in a range of specialties have indicated that PDs’ confidence in their entering residents’ preparedness to perform a range of expected activities under indirect supervision varied across specialties,^1^ and there are differences across specialties in PD expectations for their entering residents.^2,3,4^

In recent years, there has been an increased focus on the transition to residency at a national level, with many areas for potential improvement in the process highlighted in the Coalition for Physician Accountability Undergraduate-Graduate Review Committee report released in 2021.^5^ They recommended that “Early and ongoing specialty-specific resident assessment data should be automatically fed back to medical schools through a standardized process to enhance accountability and to inform continuous improvement of UME [undergraduate medical education] programs and learner handovers.”^5^ Aligned with this recommendation, the Association of American Medical Colleges (AAMC) developed the Resident Readiness Survey (RRS), a standardized instrument for PDs with PGY-1 residents in their programs to provide feedback to medical schools on their graduates’ performance in transitioning to residency.^6^ After 2 years of piloting the RRS, the RRS process was fully operationalized in 2022. Because PDs’ expectations for their incoming PGY-1 residents vary by specialty, we hypothesized that there would be differences by GME specialty at a national level in PDs’ assessments of their PGY-1 residents’ readiness for the transition to residency. We conducted a retrospective study using the first 3 years of data collected through the RRS process to test this hypothesis.

## Methods

The AAMC Human Subjects Office exempted this cross-sectional study from institutional board review. This study followed the Strengthening the Reporting of Observational Studies in Epidemiology (STROBE) reporting guideline for cross-sectional studies.

Starting in 2020, all US schools granting Doctor of Medicine degrees and, in collaboration with the American Association of Colleges of Osteopathic Medicine, Doctor of Osteopathic Medicine degrees, were provided with the option, on an annual basis, to be included in the AAMC RRS process. Since its inception, the number of schools opting each year to be included in the RRS process steadily increased (77 schools in 2020,^6^ 131 schools in 2021,^6,7^ and 168 schools in 2022^7^); there was a total of 172 unique schools during this 3-year period that opted into the RRS process, as a given school may have opted to be included in the RRS process in 1, 2, or all 3 years.^6,7^ On an annual basis, 6 months after the start of PGY-1 training, the AAMC sent the RRS^8^ to eligible PDs; that is, PDs with 1 or more PGY-1 residents in their GME programs who (1) had graduated from a school that had opted in to the RRS process, (2) had an Electronic Residency Application Service acknowledgment record, and (3) had entered into an Accreditation Council for Graduate Medical Education (ACGME)–accredited GME program immediately after graduation, based on AAMC GME Track data.^9^ Both individual (resident) level and program-level data are included in the AAMC GME Track database. Eligible PDs were invited to complete RRSs on a confidential basis, identified by the name of the PGY-1 resident via AAMC GME Track.

For this cross-sectional study, we constructed a database of individual trainee-level, deidentified data for all PGY-1 residents for whom eligible PDs were invited to complete RRSs. Our study pertained only to US medical school graduates (eg, international medical graduates, comprising roughly 23% of the ACGME-accredited GME workforce,^10^ were not included). Data came from the AAMC RRS and GME Track databases. The RRS database included a deidentified research record number for the resident, graduation year, medical school attended, and RRS data as provided by their responding PD, including the item, “During the transition to GME (0-6 months of PGY-1 year), did this resident meet overall performance expectations?” (response choices included exceeded expectations, met expectations, and did not meet expectations). We also used RRS data for PD responses to each of 17 specific areas on the survey (eTable in Supplement 1).^8^

We used AAMC GME Track program data to create GME-program level variables for specialty category and program setting as follows. We created a 10-category variable for GME program specialty: family medicine, pediatrics, internal medicine, general surgery, emergency medicine, other surgical specialties, obstetrics and gynecology, psychiatry, all other (nonsurgical) specialties, and transitional year. Using responses to the AAMC GME Track Program Survey item about program setting, we created a 4-category variable: university; community-based, university-affiliated; community-based; and other (eg, military) or unknown. At the resident-level, we used AAMC GME Track program data for program institutional affiliation and AAMC RRS database information for medical school attended to create a PGY-1 resident variable for institutional retention for GME, identifying PGY-1 residents who entered GME positions at institutions affiliated with the one that they had attended for undergraduate medical education (UME). We also included a PGY-1 year variable to account for differences in the outcome by survey year.

### Statistical Analysis

We merged all deidentified records into a single file of individual (PGY-1)-level data. We considered PD participation on a within-year basis across the 3 years of the study because each year, PD eligibility to participate was determined based on the composition of their PGY-1 cohort. Our outcome was PD response to the item “During the transition to GME (0-6 months of PGY-1 year), did this resident meet overall performance expectations?” As a major focus in the transition to residency is ensuring that every incoming resident is adequately prepared for the responsibilities they will assume at the start of the PGY-1 year, we combined met and exceeded overall expectations into a single reference group and identified the aforementioned variables independently associated with did not meet overall expectations.

We used χ^2^ tests of association for bivariate analyses and logistic regression for multivariable analysis. For residents rated as not meeting overall expectations, we tabulated for each of the 17 areas the percentage within each of 4 categories: failed to meet expectations, met expectations, exceeded expectations, and no rating (not enough information to determine, area not applicable to the program, or PD did not respond). Analyses were performed using Stata, version 18 (StataCorp LLC). A 2-sided *P* < .05 was considered statistically significant.

## Results

Table 1 gives the program characteristics of all eligible PDs invited to respond annually, grouped by response status. During the 3 years of the study, PDs received RRSs for 51 326 PGY-1 residents. Table 2 gives the characteristics of these residents grouped by RRS data availability. Our final study sample included all graduates for whom PDs provided RRS data, including the main outcome item: “During the transition to GME (0-6 months of PGY-1 year), did this resident meet overall performance expectations?” The final study sample of 29 461 PGY-1 residents with RRS data included 57.4% of all 51 326 PGY-1 residents for whom PDs were invited to complete RRSs. There were 21 865 residents excluded from the final study sample, including 21 610 for whom PDs did not provide any RRS data and 255 for whom PDs provided some data on the RRS but did not respond to the main outcome item. Inclusion in the final study sample varied by each PGY-1 training year (range, 5516 of 10 712 [51.5%] for 2020-2021 to 14 347 of 22 242 [64.5%] for 2022-2023; *P* < .001), PGY-1 program specialty category (range, 6462 of 14 603 [44.3%] for internal medicine to 3228 of 4695 [68.8%] for emergency medicine; *P* < .001), and PGY-1 program setting (range, 333 of 637 [52.3%] for other or unknown to 9466 of 15 910 [59.5%] for community-based, university-affiliated; *P* < .001) but not by resident GME institutional retention status (6744 of 11 617 [58.1%] for yes vs 22 717 of 39 709 [57.2%] for no; *P* = .09).

**Table 1.** Eligible Program Directors Invited to Respond, Grouped by Response Status

Variable	Responded, No. (%)^a^	Did not respond, No. (%)^a^	Total, No. (%)	*P* value^b^
Total	6728 (67.9)	3184 (32.1)	9912 (100)	
Survey administration year (assignment with PGY-1 y)				
2020 (2020-2021)	1786 (62.7)	1061 (37.3)	2847 (28.7)	<.001
2021 (2021-2022)	2107 (61.9)	1299 (38.1)	3406 (34.4)	
2022 (2022-2023)	2835 (77.5)	824 (22.5)	3659 (36.9)	
PGY-1 program specialty category				
Emergency medicine	536 (73.9)	189 (26.1)	725 (7.3)	<.001
Family medicine	1089 (70.3)	460 (29.7)	1549 (15.6)	
General surgery	552 (65.5)	291 (34.5)	843 (8.5)	
Internal medicine^c^	959 (64.5)	527 (35.5)	1486 (15.0)	
Obstetrics and gynecology	493 (68.4)	228 (31.6)	721 (7.3)	
Other nonsurgical specialties^d^	815 (66.5)	411 (33.5)	1226 (12.4)	
Other surgical specialties^e^	1183 (67.0)	582 (33.0)	1765 (17.8)	
Pediatrics	388 (77.3)	114 (22.7)	502 (5.1)	
Psychiatry	444 (66.4)	225 (33.6)	669 (6.7)	
Transitional year	269 (63.1)	157 (36.9)	426 (4.3)	
Program setting				
University	3155 (70.1)	1348 (29.9)	4503 (45.4)	<.001
Community based, university affiliated	2505 (67.0)	1236 (33.0)	3741 (37.7)	
Community based	989 (64.8)	538 (35.2)	1527 (15.4)	
Other or unknown	79 (56.0)	62 (44.0)	141 (1.4)	
No. of surveys sent to program director (ie, No. of eligible residents in program)				
1	1280 (62.2)	779 (37.8)	2059 (20.8)	<.001
2-4	2930 (68.4)	1353 (31.6)	4283 (43.2)	
5-9	1663 (70.6)	691 (29.4)	2354 (23.7)	
≥10	855 (70.3)	361 (29.7)	1216 (12.3)	

Abbreviation: PGY-1, postgraduate year 1.

^a^ Percentages are based on denominators in the column showing totals.

^b^ Bivariate associations were assessed by χ^2^ tests of association.

^c^ Internal medicine and internal medicine–pediatrics.

^d^ All other nonsurgical specialties (eg, dermatology, neurology, pathology–anatomic and clinical, and physical medicine and rehabilitation).

^e^ Other surgical specialties included neurological surgery, orthopedic surgery, otolaryngology, plastic surgery, thoracic surgery, urology, and vascular surgery.

**Table 2.** PGY-1 Residents Grouped by Inclusion in Final Study Sample

Variable	Residents included, No. (%) (n = 29 461)^a^	Residents excluded, No. (%) (n = 21 865)^a^	Total, No. (%) (n = 51 326)	*P* value^b^
Survey administration year (assignment with PGY-1 y)				
2020 (2020-2021)	5516 (51.5)	5196 (48.5)	10 712 (20.9)	<.001
2021 (2021-2022)	9598 (52.2)	8774 (47.8)	18 372 (35.8)	
2022 (2022-2023)	14 347 (64.5)	7895 (35.5)	22 242 (43.3)	
PGY-1 program specialty category				
Emergency medicine	3228 (68.8)	1467 (31.2)	4695 (9.1)	<.001
Family medicine	3775 (66.0)	1944 (34.0)	5719 (11.1)	
General surgery	2344 (55.5)	1882 (44.5)	4226 (8.2)	
Internal medicine^c^	6462 (44.3)	8141 (55.7)	14 603 (28.5)	
Obstetrics and gynecology	1739 (64.3)	966 (35.7)	2705 (5.3)	
Other nonsurgical specialties^d^	2822 (61.2)	1788 (38.8)	4610 (9.0)	
Other surgical specialties^e^	2581 (63.0)	1516 (37.0)	4097 (8.0)	
Pediatrics	3140 (64.7)	1713 (35.3)	4853 (9.5)	
Psychiatry	1888 (59.7)	1274 (40.3)	3162 (6.2)	
Transitional year	1482 (55.8)	1174 (44.2)	2656 (5.2)	
PGY-1 program setting				
University	16 608 (56.0)	13 024 (44.0)	29 632 (57.7)	<.001
Community based, university affiliated	9466 (59.5)	6444 (40.5)	15 910 (31.0)	
Community based	3054 (59.3)	2093 (40.7)	5147 (10.0)	
Other or unknown	333 (52.3)	304 (47.7)	637 (1.2)	
Institutional retention for GME				
Yes	6744 (58.1)	4873 (41.9)	11 617 (22.6)	.09
No	22 717 (57.2)	16 992 (42.8)	39 709 (77.4)	

Abbreviations: GME, graduate medical education; PGY-1, postgraduate year 1.

^a^ Percentages are based on denominators in the column showing totals.

^b^ Bivariate associations were assessed by χ^2^ tests of association.

^c^ Internal medicine and internal medicine–pediatrics.

^d^ All other nonsurgical specialties (eg, dermatology, neurology, pathology–anatomic and clinical, and physical medicine and rehabilitation).

^e^ Other surgical specialties included neurological surgery, orthopedic surgery, otolaryngology, plastic surgery, thoracic surgery, urology, and vascular surgery.

As is evident in Table 1, the number of PDs invited to respond to the RRS increased annually. The within-year PD RRS response rate across all 3 years combined was 6728 of 9912 (67.9%). This percentage varied by survey administration year (1786 of 2847 [62.7%] for 2020-2021, 2107 of 3406 [61.9%] for 2021-2022, and 2835 of 3659 [77.5%] for 2022-2023; *P* < .001), specialty category (range, 269 of 426 [63.1%] for transitional year to 388 of 502 [77.3%] for pediatrics; *P* < .001), and program setting (range, 79 of 141 [56.0%] for other or unknown to 3155 of 4503 [70.1%] for university; *P* < .001). PD response rates were higher as the number of surveys sent to the PD increased (from 1280 of 2059 [62.2%] for PDs who received 1 survey to 855 of 1216 [70.3%] for PDs who received at least 10 surveys). The mean (SD) RRS completion rate by responding PDs (ie, percentage of all RRSs sent to responding PDs [6728] that were completed) was 92.6% (23.5%).

Table 3 gives the characteristics of all 29 461 PGY-1 residents in the final sample grouped by outcome. Most residents in the sample had met or exceeded overall expectations in the transition to residency (28 527 [96.8%]); only 934 (3.2%) had not done so. The percentage that did not meet expectations varied by PGY-l training year (range, 137 of 5516 [2.5%] for 2020-2021 to 354 of 9598 [3.7%] for 2021-2022; *P* < .001), specialty category (range, 11 of 1482 [0.7%] for transitional year to 235 of 3775 [6.2%] for family medicine; *P* < .001), PGY-1 program setting (range, 7 of 333 [2.1%] for other or unknown to 131 of 3054 [4.3%] for community-based; *P* < .001), and PGY-1 GME institutional retention status (165 of 6744 [2.4%] for retained vs 769 of 22 717 [3.4%] for not retained; *P* < .001).

**Table 3.** Final Study Sample Of PGY-1 Residents Grouped by Program Director Response to the Resident Readiness Survey Item “During the Transition to GME (0-6 Months of PGY-1 Year), Did This Resident Meet Overall Performance Expectations?”

Variable	Expectation		Total, No. (%) (n = 29 461)	*P* value^a^
	Residents met or exceeded, No. (%) (n = 28 527)	Residents did not meet, No. (%) (n = 934)		
Survey administration year (assignment with PGY-1 y)				
2020 (2020-2021)	5379 (97.5)	137 (2.5)	5516 (18.7)	<.001
2021 (2021-2022)	9244 (96.3)	354 (3.7)	9598 (32.6)	
2022 (2022-2023)	13 904 (96.9)	443 (3.1)	14 347 (48.7)	
PGY-1 program specialty category				
Emergency medicine	3137 (97.2)	91 (2.8)	3228 (11.0)	<.001
Family medicine	3540 (93.8)	235 (6.2)	3775 (12.8)	
General surgery	2215 (94.5)	129 (5.5)	2344 (8.0)	
Internal medicine^b^	6287 (97.3)	175 (2.7)	6462 (21.9)	
Obstetrics and gynecology	1662 (95.6)	77 (4.4)	1739 (5.9)	
Other nonsurgical specialties^c^	2776 (98.4)	46 (1.6)	2822 (9.6)	
Other surgical specialties^d^	2540 (98.4)	41 (1.6)	2581 (8.8)	
Pediatrics	3057 (97.4)	83 (2.6)	3140 (10.7)	
Psychiatry	1842 (97.6)	46 (2.4)	1888 (6.4)	
Transitional year	1471 (99.3)	11 (0.7)	1482 (5.0)	
PGY-1 program setting				
University	16 176 (97.4)	432 (2.6)	16 608 (56.4)	<.001
Community based, university affiliated	9102 (96.2)	364 (3.8)	9466 (32.1)	
Community based	2923 (95.7)	131 (4.3)	3054 (10.4)	
Other or unknown	326 (97.9)	7 (2.1)	333 (1.1)	
Institutional retention for GME				
Yes	6579 (97.6)	165 (2.4)	6744 (22.9)	<.001
No	21 948 (96.6)	769 (3.4)	22 717 (77.1)	

Abbreviations: GME, graduate medical education; PGY-1, postgraduate year 1.

^a^ Bivariate associations were assessed by χ^2^ tests of association.

^b^ Internal medicine and internal medicine–pediatrics.

^c^ All other nonsurgical specialties (eg, dermatology, neurology, pathology–anatomic and clinical, and physical medicine and rehabilitation).

^d^ Other surgical specialties included neurological surgery, orthopedic surgery, otolaryngology, plastic surgery, thoracic surgery, urology, and vascular surgery.

Table 4 presents the logistic regression results. Using 2020-2021 as the baseline comparison, the odds of not meeting expectations vs meeting or exceeding expectations were higher for PGY-1 resident training in 2021-2022 (adjusted odds ratio [AOR], 1.54 [95% CI, 1.26-1.89]) and in 2022-2023 (AOR, 1.27 [95% CI, 1.05-1.54]). Using internal medicine as the reference, the odds of not meeting expectations were higher for family medicine (AOR, 2.09 [95% CI, 1.70-2.58], obstetrics and gynecology (AOR, 1.64 [95% CI, 1.24-2.15]), and general surgery (AOR, 2.05 [95% CI, 1.62-2.58]) and lower for other surgical specialties (AOR, 0.60 [95% CI, 0.42-0.84]), other nonsurgical specialties (AOR, 0.61 [95% CI, 0.44-0.85]), and transitional year (AOR, 0.22 [95% CI, 0.12-0.42]). Using university program setting as the reference, the odds of not meeting expectations were higher for community-based, university-affiliated (AOR, 1.31 [95% CI, 1.13-1.52]), and community-based (AOR, 1.42 [95% CI, 1.15-1.76]) program settings. The odds of not meeting expectations were lower for PGY-1 residents retained (vs not retained) at their home institutions for GME (AOR, 0.70 [95% CI, 0.59-0.83]).

**Table 4.** Logistic Regression Results for 934 PGY-1 Residents Not Meeting vs 28 527 PGY-1 Residents Meeting or Exceeding Expectations of 29 461 Total PGY-1 Residents

Variable	Did not meet (vs met or exceeded) expectations	
	AOR (95% CI)	*P* value
Survey administration year (assignment with PGY-1 y)		<.001^a^
2020 (2020-2021)	1 [Reference]	NA
2021 (2021-2022)	1.54 (1.26-1.89)	<.001
2022 (2022-2023)	1.27 (1.05-1.54)	.016
PGY-1 resident program specialty category		<.001^a^
Emergency medicine	0.98 (0.76-1.27)	.895
Family medicine	2.09 (1.70-2.58)	<.001
General surgery	2.05 (1.62-2.58)	<.001
Internal medicine^b^	1 [Reference]	NA
Obstetrics and gynecology	1.64 (1.24-2.15)	<.001
Other nonsurgical specialties^c^	0.61 (0.44-0.85)	.003
Other surgical specialties^d^	0.60 (0.42-0.84)	.003
Pediatrics	0.97 (0.74-1.26)	.795
Psychiatry	0.89 (0.64-1.23)	.468
Transitional year	0.22 (0.12-0.42)	<.001
PGY-1 program setting		<.001^a^
University	1 [Reference]	NA
Community-based, university-affiliated	1.31 (1.13-1.52)	<.001
Community-based	1.42 (1.15-1.76)	.001
Other or unknown	0.76 (0.36-1.63)	0.484
Institutional retention for GME		<.001^a^
No	1 [Reference]	NA
Yes	0.70 (0.59-0.83)	<.001

Abbreviations: AOR, adjusted odds ratio; GME, graduate medical education; NA, not applicable; PGY-1, postgraduate year 1.

^a^ *P* value represents a χ^2^ test for the null hypothesis that the association between the variable and the outcome in the multivariable model is equal to 0 (ie, the variable has no independent association with the outcome).

^b^ Internal medicine and internal medicine–pediatrics.

^c^ All other nonsurgical specialties (eg, dermatology, neurology, pathology–anatomic and clinical, and physical medicine and rehabilitation).

^d^ Other surgical specialties include neurological surgery, orthopedic surgery, otolaryngology, plastic surgery, thoracic surgery, urology, and vascular surgery.

The Figure shows the distribution of readiness ratings across 17 specific areas for 934 PGY-1 residents who did not meet overall expectations. The most frequently cited areas in which the PGY-1 resident failed to meet expectations were performing overall tasks and responsibilities in an organized and timely manner with appropriate attention to detail (673 of 934 [72.1%]) and prioritizing a differential diagnosis (519 of 934 [55.6%]).

**Figure.**
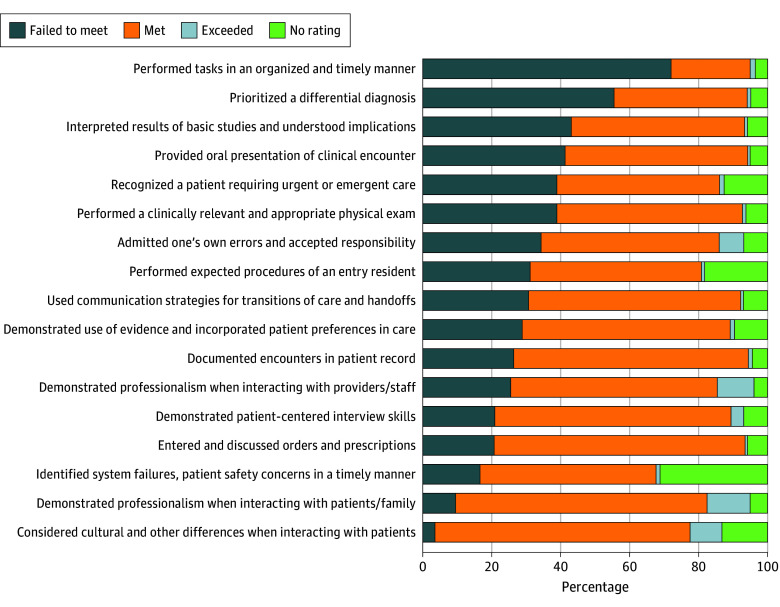
Program Director Ratings for Each of the 17 Areas Among 934 Postgraduate Year 1 Residents Who Did Not Meet Overall Expectations No rating indicates that there was not enough information to determine, was not applicable to the program director’s program, or the program director did not respond to the item.

## Discussion

The vast majority of PGY-1 residents included in this cross-sectional study met or exceeded their PDs’ overall expectations in the transition to residency. However, 3.2% of PGY-1 residents did not meet overall expectations, and their specialty category was independently associated with this outcome. Our observations regarding GME program–level and resident-level characteristics associated with PDs’ assessments of their PGY-1 residents’ readiness for the transition to residency have implications for the communities of UME and GME educators nationally.

The first year of data collected for the RRS occurred for the graduating class of 2019-2020 (PGY-1 residents in 2020-2021). This graduating class experienced minimal disruptions in their UME core medical school experiences due to the COVID-19 pandemic compared with the 2 subsequent cohorts of graduates assessed in our study.^11^ We speculate that the higher odds for PGY-1 residents in the 2 subsequent cohorts of graduates of not meeting overall expectations in the first 6 months of residency is consistent with the occurrence of the COVID pandemic regarding graduates’ preparedness for residency, particularly for the 2020-2021 graduating class (PGY-1 residents in 2021-2022). In other words, the cohorts arguably may have experienced the greatest pandemic-related disruptions throughout both their third and final years of medical school.^11,12^ However, PDs becoming more familiar with the RRS process (eg, trust in confidentiality) may have also contributed to the increase in the odds of residents not meeting expectations.

The higher response rate from PDs who received more surveys may reflect a greater appreciation among them of the value of a standardized RRS process, as these are the PDs who previously would have received a relatively larger number of school-specific surveys that were sent at different times of the year from each medical school to the PDs. The higher PD response rate in the most recent year of data collection may reflect the PDs’ growing familiarity with the process or the diminishing impact of the COVID-19 pandemic on GME programs, such that PDs may have had more time available to respond to this voluntary process.

A mix of specialty categories that were more competitive for entry into GME (on the basis of percentage of all offered positions that were unfilled in a residency matching program and percentage of filled positions that were filled by US senior applicants^13,14^), such as obstetrics and gynecology,^13^ and that were less competitive for entry into GME, such as family medicine,^13^ were among those associated with higher odds of not meeting overall expectations. Similarly, a mix of specialty categories that were more competitive for entry into GME, such as other surgical specialties,^13,14^ and that were less competitive for entry into GME, such as transitional year programs,^13^ were among those associated with lower odds of not meeting overall expectations. These observations suggest that our specialty findings were not attributable to competitiveness of the specialty per se; rather, they suggest a mismatch between how PGY-1 residents who entered GME in the specialty had prepared for the specialty and the expectations of the PDs in the specialty. Contributory factors are likely complex and vary on a specialty-specific basis, underscoring the importance of UME and GME educators working collaboratively to optimize resident readiness across all specialties.^1^

Our findings regarding specialty categories associated with greater odds of not meeting expectations may be of particular interest to PDs in these specialties as well as to UME educators preparing students to enter GME in these specialties. A recently published study of obstetrics and gynecology PDs’ and residents’ perceptions of areas in which more preparation is needed for the start of residency highlighted numerous areas of agreement in perceptions between these 2 groups and some areas of difference in perception.^4^ As these authors noted, collaboration across the UME-GME continuum is needed to ensure that all entering residents in obstetrics and gynecology can be appropriately prepared.^4^

The independent associations we observed with program setting in a model controlling for specialty category suggest that, within a given specialty category, there are differences across programs in PDs’ assessments of their PGY-1 residents. This observation is consistent with a recent study regarding milestone data reported by PDs to the ACGME in which the authors described substantial between-program variation in milestone ratings assigned by PDs for their residents in the PGY-1 year for family medicine and for emergency medicine.^15^

Over 20% of PGY-1 residents in our study had entered GME at programs affiliated with their home institutions (ie, institution affiliated with the institution the PGY-1 resident had attended for UME), and PGY-1 residents retained (vs not retained) at their home institutions for GME had lower odds of not meeting expectations. Our observation of a home-field advantage in the transition to residency extends previous observations regarding an advantage in the residency matching process.^16,17,18^ The authors of these studies noted that applicant and program preferences and priorities are likely contributory factors in graduates entering GME at their home institutions. While it may be unsurprising that PGY-1 residents who entered GME at their home institutions were generally more highly rated by their PDs than were those who did not, PDs may find this information useful as they consider postmatch preparation activities for their incoming residents, scheduling priorities and assignments during the initial months of the PGY-1 year, and resources for incoming residents entering an entirely new work environment. Our observations and those of other investigators^16,17,18^ regarding a home-field advantage in entering GME in certain specialties also have implications for equity of opportunity, as many applicants seek to enter GME in specialties for which there are no home institution GME programs at their medical schools. PDs may want to consider this in their evaluations of applications to their programs from other institutions and in their holistic review process for resident selection.^19,20^

### Limitations

Our study has numerous limitations. As our study was retrospective and observational, causality cannot be assumed. Readiness for the transition to the first 6 months of PGY-1 training may be associated with other GME variables in addition to those that we included in our study. For example, at the specialty level, the Society of Neurological Surgeons sponsors intern boot camp courses for all incoming neurological surgery PGY-1 residents at designated regional locations during the first month of GME.^21^ At the GME institution level, activities in which all incoming PGY-1 residents at a given institution (regardless of specialty) may participate during resident orientation (eg, training about institutional electronic medical records system, discharge protocols^22^) might also impact their performance during the initial months of the PGY-1 year, but any such impact probably varies widely depending on the content, duration, and educational approaches taken in such orientation activities at a particular institution.

With the initial years of RRS data collection that were included in our study, sample size limitations precluded greater stratification by specialty for some specialty categories and precluded comparison of outcomes at the individual program level within a given specialty category. These limitations could be addressed with additional years of data. Our RRS data are also limited to graduates of US medical schools.

While not a limitation per se, we did not explore UME-level variables (eg, participation in transition-to-residency courses,^23,24,25^ subinternships,^26^ time since last clinical rotation,^27^ away rotation during medical school at the program that the PGY-1 resident entered) that may be associated with resident readiness. Follow-up studies of these PGY-1 residents could be informative in examining the extent to which performance assessment data at 6 months after the start of residency may be associated with GME program retention and completion or noncompletion. Finally, it is important to acknowledge that data collection for our study occurred during a time of many other changes in the transition to residency that may impact our outcome of interest, such as the adoption of various specialty-specific approaches to program preference signaling as part of the residency application process.^28,29,30^

## Conclusions

This cross-sectional study is, to our knowledge, the first national, multispecialty study of the readiness of US medical graduates (of schools granting doctor of medicine or doctor of osteopathic medicine degrees) for PGY-1 training that used a standardized instrument across all specialties. Our findings that most PGY-1 residents met or exceeded PD expectations but that the specialty category was independently associated with readiness for the transition to residency provide the medical education community with actionable information in working across the UME-GME continuum to prepare all US medical school graduates for the transition to GME.

## Notes

Supplement 1. **eTable.** 17 Areas in the Resident Readiness Survey

Supplement 2.
Data Sharing Statement
