# Supplementary material for: Program Directors’ Assessments of US Medical Graduates’ Transition to Residency
Source: JAMA Netw Open. 2025 Jan 9;8(1):e2454048. doi: 10.1001/jamanetworkopen.2024.54048 (PMC11718550; doi:10.1001/jamanetworkopen.2024.54048)
Supplement: Supplement 1. — eTable. 17 Areas in the Resident Readiness Survey [file jamanetwopen-e2454048-s001.pdf]

Supplementary Online Content

Grbic D, Andriole DA, Roskovensky L, Speicher M, Horvath KA, Howley L. Program Directors' Assessments of US Medical Graduates' Transition to Residency. *JAMA Netw Open*. 2024;8(1):e2454048. doi:10.1001/jamanetworkopen.2024.54048

eTable. 17 Areas in the Resident Readiness Survey

This supplementary material has been provided by the authors to give readers additional information about their work.

© 2025 Grbic D et al. *JAMA Network Open*.

eTable. 17 Areas in the Resident Readiness Survey

**Survey item**	**Shorten label for figure**
Demonstrated professionalism when interacting with healthcare professionals and staff (altruism, compassion, honesty, confidentiality, and integrity).	Demonstrated professionalism when interacting with healthcare providers/staff
Demonstrated professionalism when interacting with patients and family members (altruism, compassion, honesty, confidentiality, and integrity).	Demonstrated professionalism when interacting with patients/family
Considered religious, ethnic, gender, educational and other differences in interacting with patients and other members of the health care team.	Considered cultural and other differences when interacting with patients
Admitted one's own errors and accepted responsibility for personal and professional development.	Admitted one's own errors and accepted responsibility
Performed overall tasks and responsibilities in an organized and timely manner with appropriate attention to detail.	Performed tasks in an organized and timely manner
Demonstrated patient-centered interview skills.	Demonstrated patient-centered interview skills
Performed a clinically relevant and appropriately thorough physical exam pertinent to the setting and purpose of the visit.	Performed a clinically relevant and appropriate physical exam
Performed expected procedures of an entry resident, including obtaining consent for those procedures.	Performed expected procedures of an entry resident
Prioritized a differential diagnosis.	Prioritized a differential diagnosis
Recognized a patient requiring urgent or emergent care and initiated evaluation and management.	Recognized a patient requiring urgent or emergent care
Interpreted results of basic studies and understood the implications and urgency of the results.	Interpreted results of basic studies and understood implications
Demonstrated how to access and use available evidence and incorporate patient preferences and values into delivery of care.	Demonstrated use of evidence and incorporated patient preferences in care
Documented encounters in patient record.	Documented encounters in patient record
Entered and discussed orders and prescriptions.	Entered and discussed orders and prescriptions
Provided oral presentation of clinical encounter.	Provided oral presentation of clinical encounter
Used communication strategies for safe and effective transitions of care and handoffs.	Used communication strategies for transitions of care and handoffs
Identified and reported system failures, patient safety concerns in a timely manner.	Identified system failures, patient safety concerns in a timely manner

© 2025 Grbic D et al. *JAMA Network Open*.
