# Supplementary material for: Program Directors’ Assessments of US Medical Graduates’ Transition to Residency
Source: JAMA Netw Open. 2025 Jan 9;8(1):e2454048. doi: 10.1001/jamanetworkopen.2024.54048 (PMC11718550; doi:10.1001/jamanetworkopen.2024.54048)
Supplement: Supplement 2. — Data Sharing Statement [file jamanetwopen-e2454048-s002.pdf]

Data Sharing Statement

Grbic. Program Directors' Assessments of US Medical Graduates' Transition to Residency. *JAMA Netw Open*. Published January 09, 2025. doi:10.1001/jamanetworkopen.2024.54048

Data
Data available: No

Additional Information
Explanation for why data not available: The study data are sensitive and proprietary. Access to the data was granted to the authors only for the purposes of the described study. A request for the data used in this study can be made via the AAMC Data Request form: https://www.aamc.org/request-aamc-data
